# Statistical analysis of co-occurrence patterns in microbial presence-absence datasets

**DOI:** 10.1371/journal.pone.0187132

**Published:** 2017-11-16

**Authors:** Kumar P. Mainali, Sharon Bewick, Peter Thielen, Thomas Mehoke, Florian P. Breitwieser, Shishir Paudel, Arjun Adhikari, Joshua Wolfe, Eric V. Slud, David Karig, William F. Fagan

**Affiliations:** 1 Department of Biology, University of Maryland, College Park, Maryland, United States of America; 2 Research and Exploratory Development Department, Johns Hopkins Applied Physics Laboratory, Laurel, Maryland, United States of America; 3 Center for Computational Biology, McKusick-Nathans Institute of Genetic Medicine, Johns Hopkins School of Medicine, Baltimore, Maryland, United States of America; 4 The Department of Natural Resource Ecology and Management, Oklahoma State University, Stillwater, Oklahoma, United States of America; 5 Department of Ecology, Montana State University, Bozeman, Montana, United States of America; 6 Department of Mathematics, University of Maryland, College Park, Maryland, United States of America; Partner Institute for Computational Biology Chinese Academy of Sciences and Max Planck Society, CHINA

## Abstract

Drawing on a long history in macroecology, correlation analysis of microbiome datasets is becoming a common practice for identifying relationships or shared ecological niches among bacterial taxa. However, many of the statistical issues that plague such analyses in macroscale communities remain unresolved for microbial communities. Here, we discuss problems in the analysis of microbial species correlations based on presence-absence data. We focus on presence-absence data because this information is more readily obtainable from sequencing studies, especially for whole-genome sequencing, where abundance estimation is still in its infancy. First, we show how Pearson’s correlation coefficient (*r*) and Jaccard’s index (*J*)–two of the most common metrics for correlation analysis of presence-absence data–can contradict each other when applied to a typical microbiome dataset. In our dataset, for example, 14% of species-pairs predicted to be significantly correlated by *r* were not predicted to be significantly correlated using *J*, while 37.4% of species-pairs predicted to be significantly correlated by *J* were not predicted to be significantly correlated using *r*. Mismatch was particularly common among species-pairs with at least one rare species (<10% prevalence), explaining why *r* and *J* might differ more strongly in microbiome datasets, where there are large numbers of rare taxa. Indeed 74% of all species-pairs in our study had at least one rare species. Next, we show how Pearson’s correlation coefficient can result in artificial inflation of positive taxon relationships and how this is a particular problem for microbiome studies. We then illustrate how Jaccard’s index of similarity (*J*) can yield improvements over Pearson’s correlation coefficient. However, the standard null model for Jaccard’s index is flawed, and thus introduces its own set of spurious conclusions. We thus identify a better null model based on a hypergeometric distribution, which appropriately corrects for species prevalence. This model is available from recent statistics literature, and can be used for evaluating the significance of any value of an empirically observed Jaccard’s index. The resulting simple, yet effective method for handling correlation analysis of microbial presence-absence datasets provides a robust means of testing and finding relationships and/or shared environmental responses among microbial taxa.

## Introduction

Identifying species correlations based on species presences or absences across multiple sites has a long history in ecology and biogeography. In general, the goal of such analyses is to classify, summarize and describe observed patterns in species co-occurrences that can then be used as a starting point for exploring ecological processes representing either causal relationships between species (e.g., mutualism, competition) or else similarities between species responses to the same sets of environmental factors (in the same or opposite direction). Pairwise interpretation of microbial diversity patterns remains central to functional analyses of microbiome diversity, and is sometimes complemented by other quantitative measures such as alpha-diversity [1,2], the firmicutes/bacteroides ratio [3], and analyses of the relative balance between harmless and harmful bacteria [4]. There are, however, both statistical issues and issues of interpretation that plague these types of analyses. As a result, a wide range of approaches have been developed [5–7], including at least 60 distinct correlation metrics [8] that differ in their variables, parameters, model structure, and underlying assumptions about the causes of correlation. Despite this, there is still no consensus among experts about the appropriateness of the different statistical tools and metrics, even in systems where these types of analyses have been used for decades [9–13].

More recently, correlation analyses have been extended from macroscale systems to microbial communities, with similar goals. Microbes exist in a complex web of mutualistic [14,15], commensalistic [16], parasitic, predatory [17] and competitive [18] ecological interactions [17]. Even more so than for macroscopic organisms, mechanistic understanding of these relationships are limited [14,19,20], making analysis of distributional data a primary means for identifying positive or negative functional relationships between taxa [21–23]. Fortunately, with recent progress in both amplicon and whole-genome sequencing (WGS), more and more microbiomes are being sampled, providing an ever-expanding database of systems that can be analyzed for taxon interactions [24–26].

Broadly speaking, correlation analyses can be performed in one of two ways: either using presence/absence (P/A) records or abundance data. In macroscopic systems, P/A analyses are often selected when, either due to cost constraints or logistics, sampling is insufficient to accurately resolve taxon abundances. Indeed, in cases where abundance estimates are only roughly known, P/A analysis is often found to perform best, even though it does not incorporate all available information [27]. In microbial studies, abundance data can also be problematic, though for somewhat different reasons. First, if sequencing depth is low, abundances can be difficult to resolve, particularly for rare taxa (note that this is similar to sampling effort in macroscale systems). Second, because microbial systems are sampled through sequencing, abundance estimates are necessarily relative, rather than absolute. This can generate spurious correlations [28], which have been discussed extensively in a number of recent papers proposing potential correction approaches [29,30]. Third, and most difficult to accommodate, is the uncertainty regarding abundance estimation itself [31].

Even for well-established amplicon sequencing, questions remain regarding interpretation of taxon abundances. This is a result of many factors, including (a) variability in 16S copy number [32,33], even among strains of the same species [34,35], (b) variability in 16S sequences, including within a single genome [34–36], (c) high similarity among 16S sequences from certain closely related taxa [35], (d) PCR primer mismatch [37], and (e) sequencing and taxon classification error [38]. For WGS, which is not as well-established or standardized as amplicon sequencing, the problem is even worse. First, in WGS, classified reads derive from full microbial genomes. Depending on the bioinformatics approach, this can amplify issues with incorrect read assignment, particularly when samples contain many uncharacterized taxa. Second, unlike amplicon sequencing, WGS reads are not restricted to bacteria, or even prokaryotes. Often, however, larger eukaryotic genomes are not included in reference databases, even for microbiome samples where DNA from such taxa is likely to occur. Again, this can lead to classification errors that can substantially alter abundance predictions. Third, even more so than for 16S sequencing, many of the reads generated through WGS are shared among taxa. Abundance estimates must, as a result, rely on assumptions for partitioning these reads among candidate organisms [39]. Particularly when samples contain many closely related taxa, incorrect partitioning can impede efforts to accurately predict abundances, especially at lower taxonomic levels [40]. Despite these complications, achieving correlation analyses of WGS data is imperative because it will provide better understanding of microbial interactions, especially at the species and strain level [29].

Given the current limitations on abundance estimation from WGS data, it is not surprising that relatively few correlation analyses have used WGS data [but see [41] as an example where normalized read counts were used in place of abundances]. We suggest that, as in macroecological systems with problematic abundance measurements, one potential solution for analyzing WGS is to focus on P/A data. Although this approach will not circumvent all of the difficulties associated with WGS, P/A analyses can provide more accurate determination of taxon correlations in the absence of high quality abundance estimates or when abundance data are uncertain. P/A analysis may, for example, be especially effective for systems where different abundance estimation pipelines give different results [40] or where sequencing depth is low. Unfortunately, however, research has not yet determined which P/A correlation metrics are best for analysis of different types of microbiomes or even microbiomes in general.

When P/A correlation metrics differ in their predictions, it is as a result of spurious predictions from one or both metrics being compared. Although there are several causes of spurious predictions, by far the most important is the use of metrics that fail to reflect the main processes generating correlations in the focal system. Pearson’s correlation coefficient for binary data, for example, is one of the most commonly used P/A metrics in both micro- and macroscale studies. A key assumption of this metric, however, is that sites where two species are both absent (so-called ‘co-absent sites’) feature habitats where neither species can survive [17]. However, depending on the system, co-absent sites may instead reflect locations where dispersal limitation has restricted colonization by one or both species, irrespective of habitat suitability [42]. Thus, for systems in which co-absences may be the result of factors beyond habitat suitability or requirements for mutualistic partners, using Pearson’s correlation to interpret taxon interactions can lead to faulty conclusions.

In this paper, we analyze a WGS skin microbiome dataset [26] to compare two of the most common P/A correlation metrics–Pearson’s correlation coefficient and Jaccard’s index. Strikingly, our analysis shows divergent predictions from these two popular metrics, particularly for rare taxa. Based on this finding, we discuss issues with Pearson’s correlation coefficient. Specifically, we show how Pearson’s correlation is extremely sensitive to the relative frequency of co-absent sites and how this might be a substantial problem in microbiome analysis. Our conclusion is that Jaccard’s index, which is insensitive to co-absent sites, may be a more appropriate metric for quantifying correlations in microbial systems.

However, the standard, widely used null model for Jaccard’s index [6] inflates false positives because it makes incorrect assumptions regarding species prevalence (i.e., the fraction of sites occupied). In particular, this standard null model assumes 50% prevalence for all taxa, which is clearly non-biological. Because of this assumption, the standard null model does not do a fair job of determining species correlations when species prevalences deviate strongly from 50%. Given that most biological communities feature log series or lognormal species abundance distributions, deviations from 50% occupancy are broadly expected. This has been reported for many macro-ecological systems [43], and is particularly true for microbiome datasets, which are even more likely to have distributions with long tails of rare species [44]. Instead of relying on the standard null model for Jaccard’s index, we suggest using a recently developed hypergeometric null model for species-coccurrence analysis that specifically corrects for expected changes in Jaccard’s index due to species prevalence [45].

## Materials and methods

### Input data

All whole genome shotgun data from the NCBI Sequence Read Archive (SRA) project SRP002480 were obtained from the SRA FTP site and converted to paired-end FASTQ format using the splitsra script in our Git repository hosted at the following address: https://bitbucket.org/skinmicrobiome/metagenomics-scripts. FASTQ data originating from the same BioSample were consolidated into the same file using a custom shell script and the SRA RunInfo table found here: http://www.ncbi.nlm.nih.gov/Traces/study/?acc=SRP002480.

### Reference Kraken database

A reference database was constructed for the Kraken classifier [46] using the complete genomes in RefSeq for the bacterial (2,199 taxonomic IDs), archaeal (165 taxonomic IDs), and viral (4,011 taxonomic IDs) domains, as well as eight representative fungal taxonomic IDs, the *Plasmodium falciparum* 3D7 genome, the human genome, and the UniVec Core database (ftp://ftp.ncbi.nlm.nih.gov/pub/UniVec). Low complexity regions of the microbial reference sequences were masked using the dustmasker program with a DUST level of 20 [http://www.ncbi.nlm.nih.gov/pubmed/16796549]. After masking, every 31-mer nucleotide sequence present in the collection of reference FASTA sequences was stored at the taxonomic ID of the lowest common ancestor among the leaf nodes that share that 31-mer (see [46] for details). The total size of the database plus index was 110 GB.

### Metagenomics classification

Each input read from SRA project SRP002480 was assigned a taxonomic ID using Kraken by finding exact matches between every 31-mer nucleotide sequence present in that read and the database of 31-mers constructed above. Because of the hierarchical storage of k-mers in the database, reads can be classified at more general taxonomic levels than the specific strain sequences that were used to build the database. Output from the Kraken classification was summarized by taxonomic ID along with the number of unique k-mers detected in the data using the kraken-report-modif script (present in the metagenomics-scripts repository linked above). The total number of unique k-mers for each taxonomic ID in the database was obtained using the count_kmers.pl script, and full taxonomic strings were generated using the taxid2taxstring script, both included in the metagenomics-scripts git repository linked above.

### Thresholding

Because many of the classifications based on low numbers of read counts may be spurious and/or may represent incorrect taxonomic assignments, we thresholded the data. In particular, we counted a species as present within a sample only if >100 read counts in the sample were assigned to that species. We have found that 100 reads represents a good trade-off between false negatives and false positives, although results are not particularly sensitive to this threshold.

### Pearson’s correlation coefficient versus Jaccard’s index

**Fig 1:**
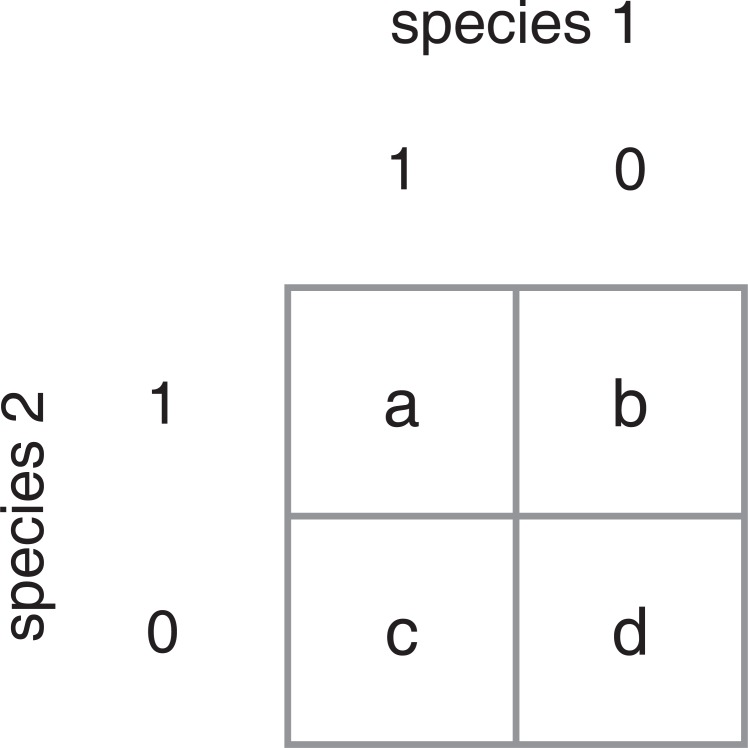
If 1 and 0 represent present and absent states of a species, this yields four possible combinations of these states for two species: co-presence (*a* in figure), mutual-exclusion (*b* and *c*), co-absence (*d*).

Pearson’s correlation for binary data, which is also known as Pearson’s product moment correlation, and is analytically equivalent to a phi coefficient [47], is a common metric for estimating association between taxa based on P/A data. A second popular metric is Jaccard’s Index (Table 1). If 1 and 0 represent present and absent states of a species, and *a*, *b*, *c* and *d* represent counts of the combinations of these states for two species as in Fig 1, then Pearson’s correlation coefficient is defined as [48]

$$r=\frac{ad-bc}{\sqrt{\left(a+b)(c+d)(a+c)(b+d\right)}}$$ (Eq 1)

while Jaccard’s index of similarity is defined as [48]

$$J=\frac{a}{\left(a+b+c\right)}$$ (Eq 2)

**Table 1:** Comparison of co-absent site percentages from different macroecological studies and our current microbiome study.

Taxon	Number of Taxa	Number of Sites	Co-absent Percent (Median)	Reference
small mammals	11	14	50%	[51]
birds	93	42	52%	[51]
lizards	5	42	55%	[51]
seed plants	1815	26	58%	[52]
butterflies	335	81	64%	[53]
fish	452	13	69%	[51,54]
amphibians	104	11	73%	[54,55]
**bacteria**	**1300**	**286**	**77%**	**current study (for species)**

The values of *r* range between −1 and 1. Taxa that are positively associated in their spatial distribution have positive *r* whereas taxa that are negatively associated have negative *r*. More extreme values represent stronger association, and values near zero indicate lack of association (see [49] for the null model). In contrast, the values of *J* range between 0 and 1, with positive versus negative correlations determined based on whether observed values of *J* fall to the extreme right or left of the appropriate, prevalence-corrected null model distribution (see below) respectively.

Both *r* and *J* are used to compute association between two species at a time. Such a bivariate analysis does not tease apart the effects of other species in the system on the relationship between the focal pair (i.e., correlated variables). Methods such as partial correlation measure the correlation between two variables with the effect of other correlated variables removed [50]. However, the vast majority of microbiome network analyses still use bivariate correlation, as this is a simple and appropriate starting point for identifying potential taxon interactions. Consequently, the goal of the current study is to report disagreement between two popular metrics of correlation as a function of the frequency of co-absent sites (*d* in Fig 1).

## Results

### Correct null model of Jaccard’s index

The standard null model for Jaccard’s index [6] has the form of a binomial distribution that determines the probability of the observed frequency of co-occurrence sites *a* out of the total number of occupied sites *n* (= *a+b+c* in Eq 2) as follows:

$$P(A=a)={(}_{a}^{n})\times 0.33^{a}\times (1-0.3{3)}^{n-a}$$ (Eq 3)

The assumption of this model is that the probability of a species being present at any particular site is independent of the presence or absence of other species and is equal to 50% (i.e., there is an equal probability of any species being absent at a site). Under this assumption, (1) uncorrelated species are expected to yield equal numbers for *a*, *b* and *c*, resulting in a Jaccard’s index (Eq 2) of 0.33, (2) as a consequence, the expectation of the null model for Jaccard’s index is 0.33, with significantly lower values indicating negative association and significantly higher values indicating positive association, (3) *a*, when expressed as a fraction of *n* for any pair of uncorrelated species is 0.33, and (4) as a consequence, the probability of co-occurrence (frequency of *a*) measured as of the number of successes out of *n* trials (frequency of all sites) can be modeled with a binomial distribution (Eq 3). The assumption of 50% prevalence of a species, however, does not account for species-site relationships (i.e., species occupancy, the fraction of sites occupied by a species, or species prevalence). Indeed, using the standard null model for Jaccard’s index, two abundant species might be identified as significantly positively correlated in occurrence just because they are each present at many sites. Likewise, two rare species might be identified as significantly negatively correlated in occurrence just because they are absent from many sites. Said differently, the standard null model for Jaccard’s index often ends up testing species pairs for significant deviations from 50% prevalence, rather than testing for significant deviations from random site filling relative to one another.

**Fig 2:**
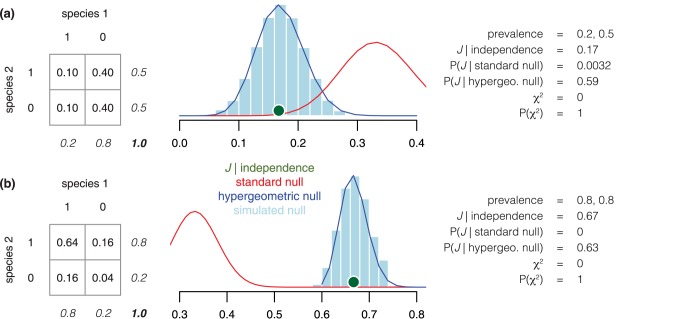
**Two examples of species pairs that are completely uncorrelated spatially that are incorrectly identified by the standard null model of Jaccard’s index** [6] **as exhibiting negative (a) and positive (b) correlation.** Probability theory indicates that two events are independent if their joint probability is the product of marginal probabilities (also indicated by Chi square statistic). In agreement with probability theory, Veech’s null model for co-occurrence analysis [45,56] and our simulated, prevalence-specific null distribution place the observed *J* right at the center of the null distribution. However, the standard null model assigns an extremely low probability for the observed *J* given the null model, making it invalid for statistical inference of *J*.

To resolve issues with the standard null model for Jaccard’s index, we must resolve (a) incorrect identification of statistically significant positive correlations when the taxa-pairs have high prevalence, (b) incorrect identification of statistically significant negative correlations when taxa-pairs are rare, (c) incorrect identification of lack of statistical significance for values of *J* near 0.33 when one or both species prevalences deviate from 50%. Correcting these issues requires a null model for *J* that takes prevalence into account. These problems can be resolved by using the null model of species co-occurrence recently developed by Veech [45]. According to this model, the null distribution of species co-occurrence takes the form of a hypergeometric distribution with parameters specific to the prevalence of the two species [56]. Specifically, the mathematical expression of the distribution for determining the probability of an observed co-occurrence between species 1 and species 2 takes the form of a classic finite population sampling problem [57] as follows:

P(X=x)=(mx)×(nk−x)(m+nk) (Eq 4)

where *m* is the frequency of sites occupied by species 1, *x* is the frequency of co-occurrence sites, *n* is the frequency of sites not occupied by species 1 and *k* is the frequency of sites occupied by species 2. Consequently, there is a separate null model of co-occurrence for each species-pair, which makes this model different from the universal standard null of Jaccard’s index developed by Real and Vargas [6]. To demonstrate the difference between the prevalence-specific null model and the standard null model, we simulate two sets of P/A data for pairs of species with independent occurences, but abundances that deviate from 50%. For both scenarios, the prevalence-specific null distribution of co-occurrence [45] includes the predicted *J* (determined for a case of independence) at the center of the distribution whereas the standard null model [6] incorrectly predicts *J* as highly significant (Fig 2). To further support the use of the hypergeometric null distribution, we generate simulated null distributions for *J* based on 100,000 trials by assuming fixed prevalences for each species, but assigning the identity of occupied sites at random. Simulated distributions very closely match Veech’s null distribution of species co-occurrence. For the remainder of the paper, we use Veech’s null model, because this provides a closed form distribution, thereby avoiding the computational time associated with simulating null distributions. We then determine positive versus negative associations in species-pairs based on whether observed *J* values lie to the right or the left of the particular hypergeometric null distribution that is specific to the prevalences of the species in each pair.

### Pearson’s correlation coefficient versus Jaccard’s index

**Fig 3:**
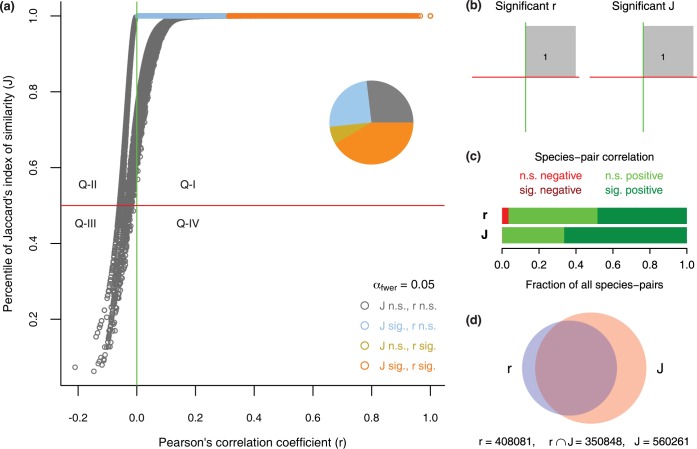
A comparison of Pearson’s correlation coefficient (*r*, also called the phi coefficient) and Jaccard’s index of similarity (*J*) for 844,350 species-pairs. (a) The similarity indices of all species-pairs, plotted in *J* by *r* plot (each pair represented by a circle), were evaluated against a familywise error rate of 5% (alpha for each hypothesis testing = 0.05/844350). Quadrant boundaries (red horizontal and green vertical lines) correspond to statistical independence for the two metrics and separate the bivariate plot into four quadrants that differ in correlation directionality. Species-pairs significant for *J* vs *r* are distinguished with different colors (“sig.” = significant; “n.s.” = not significant). All the sig. *r* but n.s. *J* pairs (gold) are hidden behind sig. *r* and sig. *J* pairs (orange). With a stringent alpha of 0.05/844350, a hard-to-notice difference in percentile of *J* makes a difference in whether it is significant or not. (b) For both *J* and *r*, all significant pairs are positive. *J* predicts 66.4% of all species-pairs to be significantly positive whereas *r* predicts only 48% significant positive. (c) Significant correlations for *r* and *J* in panel (a) are similar. The shaded regions, and the corresponding proportions, characterize the distribution of species pairs across quadrants. (d) Venn diagram illustrating that *J* and *r* selected many different species-pairs as significant, with only 56.8% of all the species pairs significant for *r* or *J* being significant for both metrics. 14% of the species pairs significant for *r* were not significant for *J* and 37.4% of the species pairs signifcant for *J* were not significant for *r*.

Fig 3 summarizes predicted correlations of all species pairs for Pearson’s and Jaccard’s indices, using the WGS skin microbiome dataset (see Materials and Methods). In general, two equally good metrics of species correlation should have high agreement: species pairs that are positively correlated in one correlation metric should be positively correlated in another metric, and vice versa. We observe the following:

*(a) match in directionality of the relationship*. Ideally, all species-pairs that are statistically significant for both *J* and *r* would fall in quadrants I and III of Fig 3A and 3B (statistical significance was evaluated against a familywise error rate of 5%; that is, alpha for each of the hypotheses tested for the 844350 unique species-pairs was 0.05/844350). Points in quadrants I and III indicate, respectively, that species predicted as being positively correlated by *r* are also predicted as being positively correlated by *J*, and that species predicted as being negatively correlated by *r* are also predicted as being negatiely correlated by *J*. Notably, we observe a perfect match in directionality between *r* and *J* for all species-pairs predicted to be significantly correlated by both metrics.

*(b) mismatch in significance*. Although both *J* and *r* predict that 100% of species-pairs significantly correlated in both metrics exhibit positive correlation (Fig 3C), *J* predicts substantially more significantly correlated taxa pairs as compared to *r* (66.4% for *J* vs 48.3% for *r*). Furthermore, a sizeable fraction of the species-pairs predicted as being significantly correlated by *r* are not predicted as being significantly correlated by *J* (Fig 3D). Specifically, 14% of species-pairs predicted to be significant by *r* are non-significant using *J*, while 37.4% of species-pairs predicted to be significant by *J* are non-significant using *r*.

### How the discrepancy between Pearson’s correlation and Jaccard’s index depends on species prevalence

**Fig 4:**
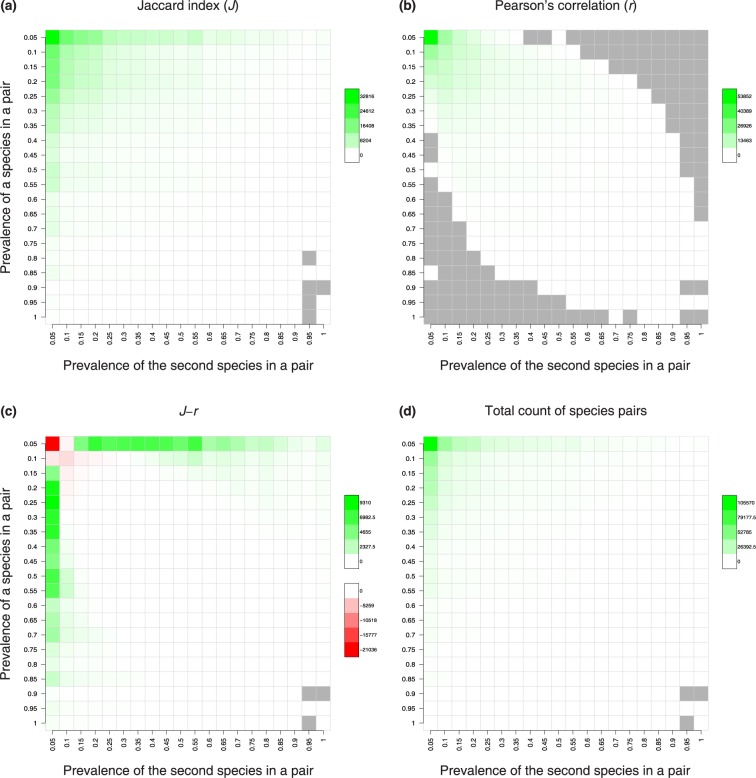
Number of species pairs identified as significant by *J* and *r* as a function of species prevalence. The prevalence of two species in a given pair are shown on the two axes of the grids. After binning the prevalence at 5% interval, the total number of pairs significant in each grid cell was counted. Color scale across plots does not match; gray cell indicate lack of species pairs. Both *J* (a) and r (b) detect many species pairs significantly correlated (all positive) when at least one of the species in the pair is rare. However, when one of the species is abundant, unlike *J*, *r* fails to detect significant pairs (b). The difference in the number of species pairs significant for *J* and *r* shows a strong pattern with species prevalence (c). Total number of species pairs in the species prevalence grid is shown in (d). Of 844350 species pairs, 627539 (74.3%) have at least one of the species in the pair very rare (<10% prevalence) whereas 205120 (24.3%) have both species very rare.

To determine why predictions for *J* and *r* deviate, we examined prediction mismatches as a function of species prevalence. This showed that when both species in the pair were moderately abundant, there was good concordance between metrics (Fig 4A–4C). However, when one or both species in the pair were rare, the two metrics diverged dramatically. This divergence was notable when at least one species in the pair exhibited a prevalence <10%, and became even more extreme when at least one of the two species exhibited a prevalence of <5%. For species-pairs with one member that was rare (prevalence <10%) and the other that was moderatly common (>20%), *r* missed many species-pairs that were identified as significant by *J* (Fig 4C, S1 Fig). By contrast, when both species in the pair were rare (<10% occupancy), *J* missed many species-pairs that were identified as significant by *r* (Fig 4C, S1 Fig).

### The problem with Pearson’s correlation coefficient

The fundamental difference between Pearson’s correlation and Jaccard’s index is that Pearson’s correlation uses co-absent sites (*d*, see Materials and Methods) to estimate taxon association. S2 Fig shows how *d*, relative to *a*, *b*, and *c*, affects *r*. When *a*, *b*, and *c* are equal, an increase in *d* always increases *r*. When *d*<*a*, *r* is negative, with less negative *r* for larger *d*. When *d*>*a*, *r* is positive, with more positive *r* for larger *d*. Hence, three different *d* scenarios give either positive or negative correlation for *r*, depending on the relative number of co-absent sites. A similar reversal of correlation direction in *r* can result even when *a ≠ b ≠ c*, for example if {*a*, *b*, *c*} = {100, 45, 65}, respectively. In this case *d* = 10 yields *r* = –0.19 and *d* = 200 yields *r* = 0.43. The sign reversal of *r* strictly as a function of the frequency of co-absent sites explains the *J*-*r* discrepancy that we observe (Fig 4C). When both species are rare, *d* is very large. This inflates *r*, making prediction of significant positive correlation more likely for this metric. However, because co-absent sites do not inflate *J*, *J* does not predict significant positive correlation for these species pairs. Therefore, there are more significant species-pairs for *r* than for *J* when both members of the species-pair are rare (Fig 4C, see S1 Fig for an interactive 3D figure). By contrast, when one species is rare and the other is relatively common, *d* is reduced relative to both *a* and *b* or *c* (depending on which of the two species is more prevalent) as compared to scenarios where both species are rare. The net result is a reduction in *r*, making it more difficult for the *r* metric to reach statistical significance. By contrast, *J* does not change as a function of *d*, while the corresponding increase in *a* inflates *J*, making prediction of significant positive correlation more likely. This is why *J* overpredicts positive correlations relative to *r* when only one member of a species-pair is rare. In the microbiome dataset we use, the *J*-*r* discrepancy has enormous impact on overall predictions of species correlations because 74.3% of all species-pairs have at least one rare species (<10% prevalence; top two rows and left two columns in the grid of Fig 4D) while 24.3% of species-pairs have both rare species (four grid cells in the top left corner of Fig 4D).

The question, then, remains whether *J* or *r* is better. Because these metrics primarily differ based on their treatment of co-absent sites, understanding the interpretations and pitfall of co-absences is crucial to selecting the best metric for a particular ecological scenario. In general, three potential issues can reduce the informative value of co-absent sites. First, problems with taxon detection can artificially inflate the number of co-absent sites (i.e., *a*, *b* or *c* sites could be classified as *d* sites), resulting in over-estimation of *r*, but having a much smaller effect on *J*. (Notice that detection problems can also result in co-present sites being classified as sites with only a single taxon present–i.e., *a* sites could be mistakenly classified as *b* or *c* sites. Although this would result in under-estimation of *r*, it would also reduce *J*, because both metrics are sensitive to reductions in *a*). Second, factors beyond habitat suitability and biotic interaction can yield taxon absences. Most obvious are dispersal constraints and stochasticity of low abundance populations. As with detection issues, these absences will have a stronger (positive) impact on *r* as compared to *J*. Third, *r* may be more sensitive to experimental design. In particular, if sampling is unintentionally biased towards co-absent sites, then this should affect *r* but not *J*. Likewise, if sites are sampled from drastically different envionments, then there will be large numbers of co-absences derived from sites that are obviously unsuitable to both species. A well-explored problem in macroecology indicates that geographic ranges for sampling absences for species distribution modeling (or, ecological niche modeling) can dramatically impact model performance. Specifically, sampling absences far from the core area of a species distribution results in poor prediction of the species distribution [58]. Disproportionately sampling co-absent sites from such environments also makes two pairs of taxa with different magnitudes of ecological association in their distributional range look more similar than they really are. This is because the information contributed by the co-present and mutually present sites (cells *a*, *b*, and *c*) of those two taxa-pairs is substantially dampened by a high frequency of co-absent sites (cell *d*).

Within the context of microbiome analysis, all three of the above issues point to using *J* in place of *r* for correlation studies. First, microbial communities are generally characterized by large numbers of rare taxa [59], making detection and stochasticity more likely to be problematic. Second, microbiomes are well-known to be under-sampled [60,61], again pointing to potential issues with detection error. Third, although the historically prevailing paradigm has been that ‘all microbes are everywhere, [and] the environment selects,’ more and more this perspective is being challenged by evidence suggesting often strong dispersal limitation among microbes [62,63]. Finally, because we lack a full understanding of the habitat requirements of most microbes, it is probable that many microbiome experiments are inadvertantly designed to sample across environments that are widely different from a microbial perspective. Because *J* ignores co-absent sites, and is thus generally more robust as compared to *r* when considering the effects of detection error, stochasticity, dispersal constraints and sampling design, we strongly recommend that researchers use *J* in analyses of microbial correlations based on P/A datasets.

## Discussion and conclusions

As more and more WGS datasets become available from a diverse array of microbial sites and habitats, having appropriate methods for characterizing microbial interactions will become more important. Because abundance estimation from WGS datasets remains problematic [40], correlation analyses based on abundances are likely to be unreliable. Consequently, we suggest P/A analyses. Historically, researchers have estimated the strength of association between two taxa from P/A datasets using Pearson’s correlation coefficient for binary outcome (equivalent to a phi coefficient). However, large numbers of non-meaningful co-absent sites caused by detection problems, stochasticity of low abundance populations, dispersal constraints, excessive sampling (which can find too many co-absent sites relative to other types of sites), and sampling from drastically different environments can make this metric problematic. Though also relevant in macroecological systems, these complications appear to be even more significant in microbial settings (see, for example, Table 1).

By contrast to Pearson’s correlation coefficient, Jaccard’s index does not consider co-absent sites. For this reason, we suggest Jaccard’s index as the metric of choice for microbiome studies. Interestingly, even in macroscale systems, the greater robustness of *J* versus *r* to sampling design and species biology has led researchers to conclude that *J* is generally superior. Hubálek [64], for example, analyzed 43 similarity coefficients of P/A data for five “admissible” theoretical criteria and concluded that Jaccard and three others indices “generally work well” for both similarity and dissimilarity. Pearson’s correlation coefficient was not one of them. Likewise, Janson and Vegelius [48] reviewed 20 metrics of correlation in P/A data for six intuitive criteria and found that *r* applied to binary data failed two criteria, whereas *J* and three other measures passed all six. Consequently, they concluded that *J* is “a very natural coefficient,” because it carries the simplest ecological interpretation [48].

One interesting question that arises from our analysis is why co-absent sites are more common in microbiome studies. There are several possible explanations. First, detection can be a bigger challenge in microbial ecology and microbiome research, where communities are highly diverse, where communities feature a large tail of rare taxa [59], and where sampling methods constitute a series of steps, each with its own error sources (e.g., sample preparation, sequencing, and bioinformatics analysis). Second, although microbes are often regarded as non-dispersal limited, recent evidence suggests that this may not be true. Consider, for instance, the skin microbiome, which we used as our example. Recently, a number of studies have shown that interpersonal contact [65–67] and/or contact with pets [66–68] can strongly influence a person’s microbiome, suggesting that dispersal opportunity plays a governing role in the microbial species recovered from different individuals. Because most human microbiome studies are performed at scales larger than the individual, dispersal limitation may explain large numbers of co-absences in P/A datasets. Alternatively, co-absences may be driven by variation in habitat suitability. Without knowing the precise habitat requirements of most microbes, it is difficult to know whether typical microbiome studies sample a broader range of microbial environments as compared to typical macroecological systems. Furthermore, imperfect understanding of microbial habitat requirements and microbial ranges means that it is also difficult to develop study designs that could potentially minimize sampling over too great a range. In macroecological studies, for example, one could address this problem by constructing a convex hull encompassing occurrences of both species, developing a union set of the convex hulls of each species, or building local convex hulls. Identification of a counterpart approach for microbial systems (based on samples taken from sites known or suspected to be within the spatial ranges of the two species) remains an open problem.

Although our analysis ultimately leads us to determine that Jaccard’s index is a more appropriate metric for P/A analysis of microbial communities, we also demonstrate that the standard null model of Jaccard’s index has some important shortcomings. In particular, Jaccard’s index inappropriately identifies some taxa pairs as signficantly correlated when, in fact, the species are spatially uncorrelated. This occurs because, historically, analyses using Jaccard’s index have tested an observed *J* against a null model that assumes 50% prevalence for all taxa. This is, in essence, a failure of the null model to account for different taxon prevalences across sampled sites. To correct for this, we suggest a recently developed null model of species co-occurrence [45] that specifically accounts for species occupancy. This method yields substantially different results as compared to historical treatments of Jaccard’s index, and improves the concordance between *r* and *J*, though there are still large discrepancies (see Results).

Although the null model that we suggest for interpretation of *J* was developed several years ago, it’s adoption by the ecology community has been slow. To demonstrate this, we reviewed all of the journal articles in English that cite [6]. This returned 41 publications, of which, ten studies determined statistical significane of *J* using a null model that was faulty. Several additional studies based conclusions on the absolute value of *J*. Importantly, this latter approach is also flawed, since *J* scores do not directly reflect correlation *without* reference to an appropriate null distribution. Indeed, as we have shown (Fig 2), the same value of *J* could indicate strong positive correlation or strong negative correlation, depending on species prevalences. Table 2 summarizes recent studies that have reported correlations between taxa or between sites based on faulty null models of *J* or without computing probabilities at all. In all studies from Table 2, evaluating *J* against the correct null model should improve statistical predictions–something that should be particularly true for cases focusing on microbial systems, where there are typically large numbers of taxa with prevalences <<50%.

**Table 2:** Examples of studies that used presence-absence data to compute Jaccard’s similarity index (*J*) for determining similarity between systems (e.g., between taxa-pairs, between sites, between markets) where the statistical significance of *J* is faulty and the use of observed value of *J* as a similarity metric is flawed.

Study	Probability of *J* determined?	Raw scores of *J* used for analysis and comparison
***Macroscopic systems***		
[71]	Not done	Sites compared based on species composition
[72]	Not done	Land use types compared based on species composition
[73]	*J* > 0.60 considered significant	Color of beach washed plastic and the one in seabird’s gut was compared to assess plastic pollution
[74]	Done with [6]	two methods for determining diet of white-tailed deer were compared based on plant species
[75]	*J* > 0.60 considered significant	Similarity in local environment plastic pollution and ingested plastic in seabirds estimated
[76]	Not done	Site similarities estimated based on *J* calculated with floristic composition
[77]	Information not available	Distributional similarity of species determined by their site-occupancy
[78]	Not done	Identity of predators was used to calculate food web similarity for many species-pairs and this this similarity was used to estimate phylogenetic signal in the community
[79]	Not done	Sites were hierarchically clustered based on *J* calculated with species composition
[80]	Not done	Sites were hierarchically clustered based on *J* calculated with species composition
[81]	Done with [6,70]	Bushmeat markets in Africa were compared for their similarity (*J*) based on the composition of taxa sold
[82]	Not done	*J* between sites determiend based on composition of plant taxa and covariates used to explain the pattern in *J*
[83]	Done with [70]	*J* between species estimated based on presence-absence in many sites
[84]	Not done	Various types of forest were compared for their similarities (*J*) based on tree species composition
[85]	Not done	Similarity of two sites (*J*) was calculated based on plant species composition
[86]	Not done	Two primate species are compared based on seed of plant species dispersed by the primates
[87]	Not done	Alpine sites were hierarchically clustered based on similarity (*J*) determined with species composition
[88]	Done with [69]	Distributional similarity between species (*J*) determiend with site-occupancy
[89]	Not done	Species-pair similarity (*J*) was determined in the environmental space
[90]	Not done	Site similarities estimated based on *J* calculated with floristic composition
[91]	Information not available	Distributional data was used to determine species-pair similarity (*J*)
[92]	Not done	Similarity between habitat types (*J*) was determined with species composition
[93]	Not done	Similarity between sites (*J*) was determined with species composition
[94]	Not done	Species-pairs compared for their similarity (*J*) in distribution
[95]	Not done	Similarity between sites (*J*) determiend with floristic composition
[96]	Done with [6]	Similarity between species (*J*) based on their distribution
[97]	Done with [70]	Similarity between sites (*J*) determiend with faunistic composition
[98]	Not done	Similarity between habitat types (*J*) determiend with species composition
[99]	*J* < 0.5 considered weak	Feed type of horses and germination of invasive species from seeds collected from fecal samples were correlated with *J*.
[100]	Not done	Site-pairs were compared for their similarity based on composition of bat species
[101]	Done with [6]	Similarity between site-pairs (*J*) based on species composition used for hierarchical clustering of sites.
[102]	Not done	Similarity between site-pairs (*J*) based on faunal composition for hierarchical clustering of sites.
[103]	Done with [6]	Similarity between geographic units based on species composition was explained by covariates
[104]	Information not available	Monthly samples of crustecean community were compared and the months were hierarchically clustered based on the similarity (*J*)
[105]	Done with [6]	Identify biogeographic divisions based on species composition similarity of various regions and the hierarchical clustering of the regions
***Microscopic systems***		
[106]	Information not available	Fungal communities associated to roots of *Cinchona calisaya* from 21 sites were compared based on presence-absence of operational taxonomic units
[107]	Not done	Various clinical and environmental isolates of *Staphylococcus aureus* were compared
[108]	Done with [6,70]	Two strains of Streptococcus pneumoniae were studied for daptomycin-sensitivity; responding genetic network was compared between the strains with *J*
[109]	Not done	Bacterial communities from two sites were compared with *J*
[110]	Not done	Similarity (*J*) in spectra of various testate amoeba found in rhizoplane of three plant *Rhododendron* species used for hierarchical cluestering
[111]	Not done	Similarity in amplification pattern of various isolates and dendrogram of hierarchical clustering

Whereas Google Scholar returns over 100,000 publications that include “Jaccard’s” or “Jaccard”, this table includes all the studies that cite Real and Vargas’s paper about the standard null model [6]. Of the 41 studies listed in this table, 24 did not determine the statistical significane of *J*, 4 lacked enough information to indicate if they determiend the statistical significance, 3 used an artibrary *J* cutoff to declare significance and 10 determined the probability but with three faulty null models: [6,69,70]. We demonstrate in Fig 2 why the most widely used null model [6] is faulty and discuss why it is faulty in the “Results” and “Discussion” sections. Two other null models for *J*, i.e. [69,70] are equally faulty because they suffer from the same problems as [6]. Irrespective of the statistical significance, comparing two observed values of *J* (as was done in every study listed in this table) is incorrect because a given value of *J* could mean anything from strong positive to strong negative correlation, depending on the species-pair specific null model (see “Results”).

We have outlined a method for identifying microbial correlations in WGS microbiome data–a task that remains under-developed in current literature. Namely we suggest using

1. P/A analysis to avoid issues with abundance estimation
2. Jaccard’s index (*J*) to circumvent problems with spurious species co-absenses
3. A prevalence-specific hypergeometric null model for *J* in order to avoid the assumption of 50% prevalence across all taxa

Specifically, we have outlined our reasons for this approach with reference to microbiome data, which are particularly prone to difficulties with abundance estimation and rare taxa. Nevertheless, many of the issues that complicate microbiome correlation analysis are also relevant to other systems in which correlation analysis based on P/A data is performed. Genomewide scanning of gene expression and other molecular studies can yield large amounts of data that likely present many of the same challenges that we have discussed. Likewise, macroecology, although less likely to suffer some of the complications associated with high biodiversity, rarity and detection errors, can still be plagued with non-informative co-absent sites, for example due to strong dispersal limitation or issues with sampling. Moreover, as field technologies improve, bringing complete sampling of diverse tropical communities within reach (e.g., LIDAR for analysis of forest canopies), we are likely to see larger and larger datasets over broader geographic regions and with increasingly automated classification pipelines similar to sequencing. In these systems, spurious correlations may also become problematic because the tendency will be to sample overly large areas containing many distinct habitats. This will artificially inflate co-absent sites (i.e., as a result of both species being very rare). In both gene expression data and macroecological systems, estimating correlations based on P/A data presents the same set of statistical problems as those discussed here. Thus, although targeted at microbial communities, we fully expect that the methodological improvements developed here should facilitate analyses in a diversity of other correlation networks as well.

## Supporting information

S1 Fig Interactive 3D graphics of Fig 4C in the main text. The file is best viewed in Google Chrome.
(HTML)

S2 Fig How the relative frequency of co-absent sites impacts Pearson’s correlation coefficient. A simulation shows how an increase in the frequency of co-absent sites ([–,–] in the occurrence matrix; denoted by red *d* relative to that of co-present and mutual-exclusion sites (*b*, *c*) inflates Pearson’s correlation coefficient, often changing the direction of correlation. Each curve shows the trajectory of change in *r* as the frequency of coabsent sites is increased for a fixed number of sites *a*, *b* and *c*. The horizontal line at *r* = 0 has the values of *r* when occurrence frequencies *a*, *b*, *c* and *d* are all equal. A decrease in *d* relative to the others results in negative *r* whereas an increase results in positive *r*. Significant negative correlations appear in red on each curve, and significant positive correlations appear in green.
(EPS)
