# Supplementary material for: Statistical analysis of co-occurrence patterns in microbial presence-absence datasets
Source: PLoS One. 2017 Nov 16;12(11):e0187132. doi: 10.1371/journal.pone.0187132 (PMC5689832; doi:10.1371/journal.pone.0187132)
Supplement: S1 Fig — The file is best viewed in Google Chrome. (HTML) [file pone.0187132.s001.html]

 
RGL model


You must enable Javascript to view this page properly.

  
Drag mouse to rotate model. Use mouse wheel or middle button
to zoom it.

---

  
Object written from rgl 0.97.0 by writeWebGL.
